# Chromosome-level genome assembly provides insights into the genome evolution and functional importance of the phenylpropanoid–flavonoid pathway in *Thymus mongolicus*

**DOI:** 10.1186/s12864-024-10202-8

**Published:** 2024-03-19

**Authors:** Zhenhua Dang, Ying Xu, Xin Zhang, Wentao Mi, Yuan Chi, Yunyun Tian, Yaling Liu, Weibo Ren

**Affiliations:** 1 Ministry of Education Key Laboratory of Ecology and Resource Use of the Mongolian Plateau & Inner Mongolia Key Laboratory of Grassland Ecology, School of Ecology and Environment, Inner Mongolia University, Hohhot, 010070 China; 2 Ministry of Education Key Laboratory of Herbage & Endemic Crop Biotechnology, School of Life Sciences, Inner Mongolia University, Hohhot, 010070 China; 3 Key Laboratory of Forage Breeding and Seed Production of Inner Mongolia, Inner Mongolia M-Grass Ecology and Environment (Group) Co., National Center of Pratacultural Technology Innovation (under preparation), Ltd, Hohhot, 010060 China

**Keywords:** *Thymus mongolicus*, Chromosome-level genome, Whole-genome duplication, Genome evolution, Phenylpropanoid–flavonoid biosynthesis, Transcriptome and metabolome

## Abstract

**Background:**

*Thymus mongolicus* (family Lamiaceae) is a *Thyme* subshrub with strong aroma and remarkable environmental adaptability. Limited genomic information limits the use of this plant.

**Results:**

Chromosome-level 605.2 Mb genome of *T. mongolicus* was generated, with 96.28% anchored to 12 pseudochromosomes. The repetitive sequences were dominant, accounting for 70.98%, and 32,593 protein-coding genes were predicted. Synteny analysis revealed that Lamiaceae species generally underwent two rounds of whole genome duplication; moreover, species-specific genome duplication was identified. A recent LTR retrotransposon burst and tandem duplication might play important roles in the formation of the *Thymus* genome. Using comparative genomic analysis, phylogenetic tree of seven Lamiaceae species was constructed, which revealed that *Thyme* plants evolved recently in the family. Under the phylogenetic framework, we performed functional enrichment analysis of the genes on nodes that contained the most gene duplication events (> 50% support) and of relevant significant expanded gene families. These genes were highly associated with environmental adaptation and biosynthesis of secondary metabolites. Combined transcriptome and metabolome analyses revealed that *Peroxidases*, *Hydroxycinnamoyl-CoA shikimate/quinate hydroxycinnamoyl transferases*, and *4-coumarate-CoA ligases* genes were the essential regulators of the phenylpropanoid–flavonoid pathway. Their catalytic products (e.g., apigenin, naringenin chalcone, and several apigenin-related compounds) might be responsible for the environmental tolerance and aromatic properties of *T. mongolicus*.

**Conclusion:**

This study enhanced the understanding of the genomic evolution of *T. mongolicus*, enabling further exploration of its unique traits and applications, and contributed to the understanding of Lamiaceae genomics and evolutionary biology.

**Supplementary Information:**

The online version contains supplementary material available at 10.1186/s12864-024-10202-8.

## Background

Plant genome contains entire genetic information of a species, recording its evolutionary history and direction. Comprehensive understanding of plant genome evolution is important in revealing the relationships and evolutionary processes among plant species. Moreover, it reveals the genetic foundations that endow plants with distinct morphological, physiological, and metabolic characteristics and provides insights into the mechanisms underlying adaptation of plants to specific environments and the development of distinct biological traits [1]. In recent years, rapid development of long-read sequencing technologies and high-throughput chromosome conformation capture (Hi-C) methods has facilitated the generation of plant genome assemblies at the chromosome level [1–4]. The high-quality reference genomes and relevant phylogenomic analysis have strongly helped in identifying the plant genome organization properties, enhanced the knowledge of plant evolution, and greatly enriched the tree of life.

Whole-genome duplication (WGD) is widespread in angiosperms and plays a crucial role in plant evolution and diversification [5, 6]. Through WGD events, tandem repeats, transposable element-mediated repeats, and retrotransposon duplications, genomes can increase in size and gene number. This results in the generation of structurally and functionally similar gene copies and formation of gene families [7]. This facilitates adaptation of plants to diverse biotic and abiotic environments and may lead to speciation. For instance, WGD events and tandem duplication collectively shaped the genomes of fern plants *Tmesipteris tannensis* and *T. obliqua* [8]. The genome of *Liriodendron chinense* has evolved through an ancient WGD event and recent repetitive sequence bursts, leading to specific patterns of floral color and petal evolution [9]. An ancient WGD event that occurred before the radiation of most Ericaceae plants contributed to the genomic architecture of flowering time [10].

Duplicated genes provide raw materials for plant gene evolution, and expansions or contractions of gene families can lead to species divergence. However, the increase in plant gene content is not directly proportional to gene duplication. Under the influence of natural selection, genetic drift, and mutation, most duplicated genes undergo processes including retention, loss, neo-functionalization, sub-functionalization, and expression divergence [11]. This allows the retention of beneficial genes, relaxation of selective pressures, and ultimately acquisition of new adaptive traits [12]. In *Chimonanthus praecox*, most genes related to floral-fragrance synthesis arose from both tandem and whole-genome duplication events [13]. In the *Angiopteris fokiensis* genome, the retained genes exhibited strong relationships with nutrient metabolism, signal transduction, and adaptive regulation [14]. The significantly expanded gene families in the genome of *Euscaphis japonica* are closely related to its strong environmental adaptability and fruit skin coloration [15].

*Thymus* is an herbaceous aromatic plant of the family Lamiaceae, known for its fragrance. This plant can be used as a food, medicine, and spice and in honey production [16, 17]. *Thyme* essential oil is rich in compounds such as phenols, terpenes, and alcohols [18] and exhibits antimicrobial, antiviral, and antioxidant activities [19, 20]. The composition of essential oil is significantly different among different *Thymus* species [21, 22]. The major components of essential oil of *T. vulgaris* include thymol (48.1%), p-cymene (11.7%), 1,8-cineole (6.7%), γ-terpinene (6.1%), and carvacrol (5.5%) [23]. In contrast, the essential oil of *T. quinquecostatus* exhibits a high content of carvacrol (30.53%) and a low content of thymol (0.46%) [24]. In the essential oils of *T. dahuricus*, *T. nervulosus*, *T. mongolicus*, and *T. quinquecostatus*, thymol has the highest content (32.86%–79.04%) with variation among species [25]. Therefore, extensive studies are needed to investigate the synthesis and metabolic processes of essential oils in different *Thymus* species to provide guidance for their development and utilization.

**Fig. 1:**
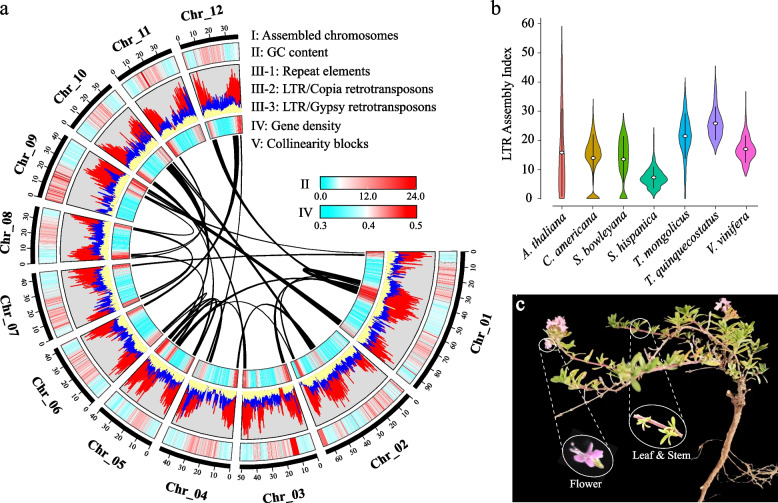
The features of *Thymus mongolicus* genome. **a** The landscape of *T. mongolicus* genome. The circus plot from the outer to the inner circle represents chromosome-scale pseudochromosomes (Chr01–Chr12) (I), GC content (II), repeat element (III-1), Ty1/copia retrotransposons (III-2), Ty3/gypsy retrotransposons (III-3), gene density (IV), and each linking line in the center of the circus plot indicates the collinearity blocks panning more than 40 genes in the genome (V). **b** LTR assembly index (LAI) analysis for the referenced genomes. **c** *T. mongolicus* plant

*T. mongolicus* (Fig. 1c) is a drought-tolerant species, characterized by well-developed root systems and strong adaptability to barren soil, drought, cold, and pests [26, 27]. *T. mongolicus* can form dominant communities in fragile and degraded environments, playing a significant ecological role in the succession of biota in desertified soil [28, 29]. Currently, research on *T. mongolicus* primarily focuses on its distribution, physiological characteristics, essential oil composition, and population genetics [30–33]. However, unavailability of whole genome information limits the extensive exploration of the genetic resources and applications of this species. In this study, using long-read sequencing and Hi-C technologies, we constructed a chromosomal-level genome of *T. mongolicus* and revealed its genomic features, evolutionary history, and phylogenetic position within the Lamiaceae family. Furthermore, combined transcriptomic and metabolomic analyses were conducted to elucidate the significant role of secondary metabolite (i.e., phenylpropanoids and flavonoids) synthesis in imparting the properties of aroma and environmental adaptation in this species. This study laid a foundation for further investigating the molecular mechanisms underlying the synthesis of specific compounds in *T. mongolicus*.

## Methods

### Sample collection

In July 2020, samples of *T. mongolicus* (leaves, stems, roots, and flowers) were collected from the eastern edge of Xilinhot (E116°06ʹ, N43°57ʹ), Inner Mongolia, China. The samples were immediately frozen in liquid nitrogen and stored at − 80℃.

### Library construction and genome sequencing

The genomic DNA of *T. mongolicus* was extracted from the leaves using a modified CTAB method [34]. Quality of the isolated DNA was assessed using NanoDrop-2000 (Thermo Fisher Scientific, Wilmington, DE, USA) and Qubit 3.0 fluorometer (Life Technologies). The genomic DNA was sheared to an average size of 500 bp, and a library with an insert size of 500 bp was prepared using the PairedEnd DNA Sample Prep kit (Illumina Inc., San Diego, CA, USA). The library was sequenced using the Illumina NovaSeq6000 platform (Illumina Inc., San Diego, CA, USA) for genome survey and assessment. For genome sequencing, DNA fragments > 20 kb were selected for library preparation using BluePippin (SAGE). The PacBio library was prepared using the SMRTbell Template Prep Kit-SPv3 following the manufacturer’s instructions (Pacific Biosciences, Menlo Park, CA, USA) and was sequenced on the Pacific Biosciences Sequel system (Pacific Bioscience, CA, USA). The Hi-C library [35] was constructed using the HindIII restriction enzyme as per the manufacturer’s instructions (BioMarker Technologies Company) [36] and was sequenced on Illumina NovaSeq6000 platform for chromosome construction. For RNA-seq used in genome assembly and annotation, libraries were constructed for the root, stem, leaf, and flower of *T. mongolicus* using a paired-end model and were sequenced on Illumina NovaSeq6000 platform.

### Estimation of genome size and heterozygosity

The raw reads obtained from Illumina sequencing were filtered using Fastp (version 0.18.0) [37] to remove reads contaminated with adapters, low-quality reads (> 50% low-quality nucleotides), and reads with ambiguous nucleotides (> 10% N). Further, jellyfish (version 2.2.6) [38] and GenomeScope (version 1.0.0) [39] were used to count k-mers and estimate the size, repetitive sequences, and heterozygosity of the *T. mongolicus* genome. The information on the peak depth and number of 17-mers was obtained based on k-mer analysis. The relationship between these factors was expressed as follows: Genome size = K-mer number/peak depth.

### Genome assembly and quality assessment

The PacBio raw subreads were filtered and corrected using the PacBio circular consensus sequencing (pbccs) pipeline with default parameters. De novo assembly of the resulting PacBio sequencing data was performed using Hifiasm software (version 0.16.1) [40]. Potential sequence errors in the initial assembly with Illumina sequencing reads were corrected using pilon (version 1.18) [41]. The quality of the Hi-C data was evaluated using the HiC-Pro pipeline [42]. Bowtie2 (version 2.2.6) [43] global was used to map the paired-end Hi-C reads to the draft assembled genome. The resulting paired-end Hi-C reads were used to cluster, orient, and link the assembled contigs into pseudochromosomes using YaHS (version 1.1) [44] and Endhic [45]. Finally, the integrity of the obtained genomes was assessed using Benchmarking Universal Single-Copy Orthologs (BUSCO) alignment (version 3.0.2) [46] and LTR assembly index (LAI) [47]. LAI score of the *T. mongolicus* genome was obtained using LTR_retriever with default parameters.

### Repeat annotation

The tandem repeats were identified using Tandem Repeats Finder (version 4.09) [48]. A combined strategy was employed for transposable element (TE) prediction. Through a de novo-based approach, RepeatModeler (version 1.05), RepeatScout (version 1.05) [49], LTR_Finder [50], and LTRharvest [51] were used to discover the TEs and build a TE library. For TE annotation based on homology, RepeatMasker (version 1.332) [52] was used to search the RepBase database (version 18.07) [53] for repetitive DNA, and RepeatProteinMasker (version 4.0.7) [54] was used to search the protein database for TE-related proteins. LTR_retriever [47] was used to combine LTR retrotransposons (LTR-RTs) in the *T. mongolicus* genome and estimate the insertion time of LTR-RTs. Subfamilies of LTR-RTs were classified using TEsorter (version 1.4.6) [55, 56]. The most representative LTR-RT elements (i.e., Ty3/gypsy and Ty1/copia retrotransposons) were aligned and trimmed using MAFFT (version 7.505) [57] and trimAl (version 1.4.rev15) [58], respectively. Further, the optimal model was identified using IQ-TREE (version 1.6.11) [59], and phylogenetic trees representing each LTR-RT family were constructed with the ML model using RAxML with 1000 bootstrap replicates (version 8.2.12) [60]. The best-obtained models for Ty3/gypsy and Ty1/copia elements were LG + G4 and JTT + F + G4, respectively. The phylogenetic trees were visualized using iTOL (version 6) [61].

### Gene prediction and functional annotation

The *T. mongolicus* genome was searched for protein homologs by aligning the protein sequences of seven homologous species (*Arabidopsis thaliana*, *Buddleja alternifolia*, *Cannabis sativa*, *Lactuca saligna*, *Populus deltoides*, *Salvia splendens*, and *T. quinquecostatus*) using Miniprot (version 2) [62]. De novo gene prediction was performed using Augustus (version 2.7) [63] and SNAP (version 2013_11_29) [64]. The resulting gene sets were merged into a nonredundant gene set using EVidenceModeler (version 1.1.1) [65]. The quality of the gene set was evaluated using BUSCO (RRID: SCR_ 015008). Furthermore, BLAST (version 2.10.0) with a threshold e-value of 1e^−5^ was used to annotate the molecular functions of predicted protein-coding genes using five databases, including the nonredundant protein sequence (Nr), Swiss-Prot, Gene Ontology (GO), Eukaryotic Orthologous Groups (KOG), and Kyoto Encyclopedia of Genes and Genomes (KEGG) databases.

### Noncoding RNA annotation

tRNA genes were identified using tRNAscan-SE (version 2.0.9) [66] with default parameters. rRNAs and their subunits were predicted using Barrnap (version 0.9). The miRNAs and snRNAs were predicted by searching sequence information against the Rfam database using Infernal cmscan (version 1.1.4) [67].

### Synteny analysis and genome duplication assessment

To determine whole-genome duplication events, intra- and inter-specific synteny analyses were performed across the genomes of *T. mongolicus* and other 7 species (*A. thaliana*, *Oryza sativa*, *Salvia bowleyana*, *Salvia hispanica*, *Callicarpa americana*, *Vitis vinifera*, and *T. quinquecostatus*). Initially, the Python scripts provided by Whole-Genome Duplication Integrated analysis (WGDI, version 0.6.2) [68] were used to generate a modified GFF file for a genome, and alternatively spliced transcripts were filtered out. Subsequently, Diamond (version 2.1.6–1) [69] was used for performing protein–protein BLAST (e-value ≤ 1e^−5^), and the results were formatted in fmt6.blast. Further, the -d, -icl, -ks, -bi, -bk, -c, and -kp commands of WGDI were successively executed with the default parameters [70]. Finally, the median *Ks* values of the collinear genes were visualized with the R package ggplot2. According to the estimated *Ks* peaks by -pf program of WGDI, WGDs and speciation events were calculated using the formula: time = *Ks*/(2 × r). The synonymous substitutions per site per year as 8.61 × 10^−9^ (r) were estimated using divergence time [71]. Chromosome karyotype analysis was performed using TBtools (version 1.108) [72] by calling one-step MCScanX-super fast and multiple synteny plot plugins, and tandem gene pairs were obtained. TBtools was used to visualize the genome landscape including pseudochromosomes, GC content, repeats, gene density, and synteny gene regions. To identify the pattern of genome-wide duplications in *T. mongolicus*, duplicated genes were divided into five categories: WGD, TD, PD, TRD, and DSD, using DupGen_Finder (version 1.12) with the default parameters [73].

### Phylogenetic analysis and estimation of divergence

The genomes of *T. mongolicus* and other 9 species (*A. thaliana*, *C. americana*, *Ocimum sanctum*, *O. sativa*, *Perilla frutescens var. Hirtella*, *S. bowleyana*, *S. hispanica*, *V. vinifera*, and *T. quinquecostatus*) were used to cluster paralogous and orthologous groups using Orthofinder (version 2.5.5) [74]. Single-copy orthologous genes were used to construct the phylogenetic tree. The alignments obtained from MUSCLE (version 3.8.31) [75] were converted into coding sequences. A new matrix was formed with Gblocks (version 0.91b) [76] by extracting the conservative regions and deleting the gap information in the sequence. The optimal model was used with IQ-TREE (version 1.6.11) [59] to analyze the tree and infer the divergence dates. Trees of 10 species were constructed with the ML model using RAxML with 1000 bootstrap replicates (version 8.2.12) [60], and the best-obtained model was GTR + F + R4. The divergence time was calculated using the MCMCtree program in PAML (version 4.9) [77] with the single-copy gene families. Time correction points were obtained from the TimeTree database. Two known divergence time points for *O. sativa* and *V. vinifera* (CI: 143.0–174.8 Mya) and grape and *A. thaliana* (CI: 109–123.5 Mya) were used. To identify the probable gene duplication events in the analyzed species, Orthofinder (version 2.5.5) with the “-M msa” parameter was used to infer gene duplications using the STRIDE method and duplication-loss-coalescence model [74]. The duplicated genes from phylogenetic branches with support values > 50% were subjected to functional enrichment analysis.

### Gene family evolution

Computational Analysis of Gene Family Evolution (CAFE) (version 5.0) [78] with default parameters was used to identify the expansions and contractions of gene families following divergence predicted by the phylogenetic tree with the predicted divergence time of the analyzed species. An Orthofinder-generated gene count list was used to perform CAFE analysis. Conditional P value was calculated for each gene family, and families with conditional *P* < 0.05 were considered to have had a significantly accelerated rate of expansion or contraction. Subsequently, functional enrichment analysis of the genes of the significantly expanded and contracted gene families was performed.

### Transcriptome sequencing and bioinformatic analysis

Total RNA was extracted using the Qiagen RNAeasy kit (Qiagen, Hilden, Germany) as per the manufacturer’s instructions. RNA quality was confirmed using the Fastp [37]. Sequencing libraries of root, stem, leaf, and flower tissues were constructed and sequenced on the HiSeq 2500 Illumina sequencing platform to generate paired-end reads with a length of 150 bp.

The raw reads were processed using Trimmomatic (version 0.32) [79] to remove adapter sequences and low-quality reads to obtain clean reads. To map the paired-end clean reads to the assembled *T. mongolicus* genome, an index was constructed using Bowtie2 (version 2.2.6) [43]. The alignment was performed using HISAT2 (version 2.2.1) [80] with the parameter “rna-strandness RF.” The expression levels of transcripts in each sample were quantified using RSEM (version 1.3.3) [81], and fragments per kilobase of transcript per million mapped reads (FPKM) was calculated. Differential expression analysis was performed using DESeq2 (version 1.22.1) [82], with criteria |log2 fold change (FC)|≥ 1 and false discovery rate ≤ 0.05 to identify significant differentially expressed genes (DEGs) between the two compared tissues.

### Metabolomic assay

The freeze-dried samples of four different tissues of *T. mongolicus* were ground with zirconia beads at 30 Hz using a mixer mill (MM 400; Retsch, Haan, Germany) for 1.5 min. Each 100 mg sample was extracted overnight at 4 °C with 1: 1 methanol: water on a rotating wheel. After centrifugation at 10,000 *g* for 10 min, the supernatants were collected and filtered. UPLC-ESI–MS/MS analysis was performed using an ACQUITY UPLC HSS T3 C18 chromatographic column [83]. For the solvent system, phase A was 0.1% formic acid in ultrapure water and phase B was 0.1% formic acid in acetonitrile. The conditions were as follows: injection volume 5 µL, flow rate 0.4 mL/min, and column temperature 40 °C. The raw data were qualitatively and quantitatively analyzed using Analyst software (version 1.6.3). Differential metabolites were identified using principal component analysis, FC, and orthogonal partial least squares-discriminant analysis, with the criteria FC > 1 or < − 1, *P* < 0.05. The identified metabolites were annotated based on the KEGG compound database and mapped to the KEGG pathway database. Volcano plots were used to filter the metabolites of interest based on the log2 (FC) and log10 (P) of metabolites. Further, metabolic pathway enrichment analysis of the differential metabolites was performed.

### Regulation of the genes from phenylpropanoid–flavonoid (PF) pathway

The correlation analysis was conducted using Pearson’s correlation coefficient in the R package to calculate the correlation coefficient Mantel’s *r* and *P* value (Mantel’s *P*) of genes and metabolites in the PF pathway. To identify the regulatory relationships of gene expression in the PF pathway, transcription factors (TFs) in the *T. mongolicus* were integrated into a weighted gene co-expression network analysis using the OmicShare tool. The co-expressed modules were attained with the following parameters: soft threshold power = 14, TOMtype = signed, mergeCutHeight = 0.25, and minModuleSize = 50. The networks between genes and TFs were visualized in Cytoscape (version 3.7.1) [84].

## Results

### Genome sequencing and assembly

The k-mer analysis revealed that the estimated genome size of *T. mongolicus* was 559.25 Mb, with 1.99% heterozygosity rate and 57.64% repetitive sequences (Table S1). Using Illumina, PacBio, and Hi-C sequencing technologies, 12.01 Gb Illumina reads, 56.5 Gb PacBio long reads, and 47.53 Gb Hi-C reads were obtained, respectively (Table S2). The total length of the assembly was 605.2 Mb, with contig N50 and scaffold N50 sizes of 3.96 and 35.95 Mb, respectively (Table 1). Based on the Hi-C interaction maps (Fig. S1), 582.7 (96.28%) Mb of the scaffold was anchored onto 12 chromosomes (Fig. 1a and Table S3). The Benchmarking Universal Single-Copy Orthologues (BUSCO) analysis indicated that 97.58% (1,575) of the core genes were complete in the assembled *T. mongolicus* genome (Fig. S2 and Table S4). Long terminal repeat (LTR) annotation revealed an LTR assembly index (LAI) score of 20.31 (Fig. 1b) of the assembled *T. mongolicus* genome, which reached the gold quality.

**Table 1:** Major characteristics of the *T. mongolicus* genome assembly and annotation

Genome assembly	Number/Size
Genome size (Mb)	605.2
Number of scaffolds	1411
N50 scaffold length (bp)	35,951,968
N90 scaffold length (bp)	111,941
Number of contigs	1585
N50 contig length (bp)	3,963,684
N90 contig length (bp)	111,478
Genome annotation	
Protein-coding genes	32,593
Average gene length (bp)	3647.26
Average CDS length (bp)	1401.19
Average exon length (bp)	1719.82
Average intron length (bp)	2051.94
Average mRNA per gene	1.21
Average mRNA length (bp)	3771.76

### Repeats and gene annotation

Approximately 430.40 Mb repetitive sequences were identified in the *T. mongolicus* genome (Table S5). Among them, LTR retrotransposons (353.76 Mb) were the most abundant, followed by tandem repeats and DNA transposons. The other types of repeat sequences accounted for a relatively small proportion (Table S5). Ty3/gypsy and Ty1/copia exhibited the highest proportion in the transposable elements (Table S6). Additionally, the repeat elements were unevenly distributed on the chromosomes and highly associated with the gene density on each chromosome (Fig. 1a). Overall, 32,593 protein-coding genes were predicted, with an average gene length of 3,647.26 bp (Table 1 and S7). BUSCO analysis revealed that 98.51% of the annotated genes were completely assembled (Table S8). Of the predicted genes, 29,989 (92.01%) were functionally assigned to the public database (Table S9). Additionally, 15,676 non-coding RNAs (ncRNAs) were identified, including 8,066 rRNA, 2,295 miRNA, 1,309 snRNA, and 4,006 tRNA (Table S10).

### Whole-genome duplication and synteny analysis

**Fig. 2:**
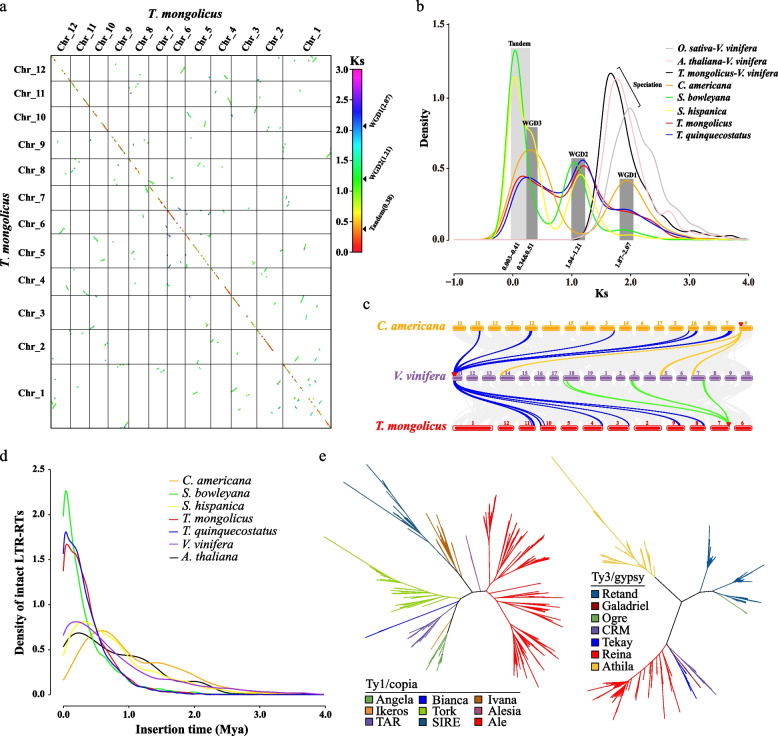
Evolution of *T. mongolicus* genome. **a** The dot plot of synteny blocks of *T. mongolicus.* **b** Intra- and inter-specific *Ks* densities of the identified synteny blocks in the analyzed genomes. **c** Genomic karyotype analysis of *T. mongolicus*, *V. vinifera*, and *C. americana.* Gray bands in the background indicate synteny blocks between the genomes. Some 1: 6 and 3: 1 blocks (*V. vinifera* vs. *Thymus* plants) are highlighted. **d** Estimation of the insertion of LTR retrotransposons in the analyzed genomes. **e** Clustering analysis of the Ty3/gypsy and Ty1/copia retrotransposons in the *T. mongolicus* genome

In *T. mongolicus* genome, most syntenic regions exhibited 1:1 relationship (green lines), whereas a minority of blue collinearity blocks exhibited 1:2 relationship (Fig. 2a). Between *T. mongolicus* and *Vitis vinifera* genomes, the collinearity relationship was mainly 1:3 (Fig. 2c and S3). In the opposite comparison, the two genomes were predominantly in 1:2 relationship, and the syntenic ratio depth varied from 1 to 6. For *C. americana*, 1:1 relationship was obvious among the golden syntenic blocks; additionally, the blue syntenic blocks with 1:2 relationship were detected (Fig. S3).

Synonymous substitution rate (*Ks*) distribution revealed a peak ranging from 1.87 to 2.07 in the Lamiaceae family. After the divergence of grape and *Thyme*, the *Ks* values peaked at 1.04–1.21 for *S. bowleyana*, *S. hispanica*, *T. mongolicus*, and *T. quinquecostatus* and at 0.34 and 0.51 for *C. americana* and *S. hispanica*, respectively (Fig. 2b). Based on the divergence time estimates, we speculated that the Lamiaceae species sharing two *Ks* peaks may represent a WGT, and a following WGD happened at approximately 108.59–120.21 and 60.39–70.27 Mya, respectively. The two specific peaks for *C. americana* and *S. hispanica* indicated that species differentiation occurred at 19.74 and 29.62 Mya, respectively. In the most recent period of 0.17–23.8 Mya (Ks = 0.003–0.41), different levels of insertion of repetitive sequences (i.e., tandem and interspersed repeats) may have occurred in the genomes of these species (Fig. 2b). LTR analysis revealed numerous LTR insertions in the genomes of Lamiaceae species in the recent period (< 2 Mya), particularly in the two *Thymus* species and *S. bowleyana* (Fig. 2d and S4). In *T. mongolicus*, AIe and Reina were the most predominant clade LTR-TRs in the Ty1/copia and Ty3/gypsy families (Fig. 2e).

### Phylogenomic and gene family analyses

**Fig. 3:**
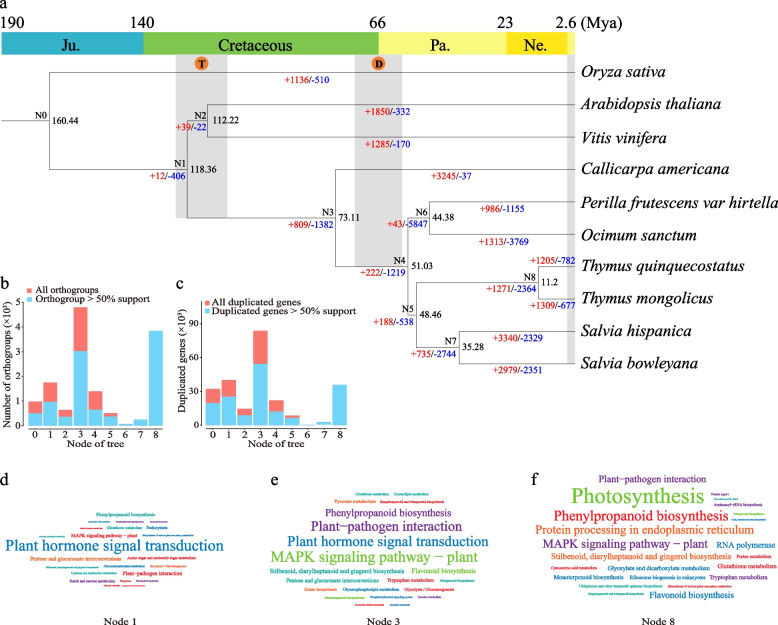
Genome evolution and gene family analysis. **a** A phylogenetic tree was constructed based on 658 high-quality single-copy orthogroups from 10 plant species. Regions T and D represent the time of WGT and WGD in the species, respectively. The numbers of gene-family expansion and contraction on each branch are indicated by red and blue numbers, respectively. **b** Statistics of orthogroups on each node. Red represents all orthogroups. Blue represents orthogroups with > 50% support. **c** Statistics of duplicated genes on each node. Red represents all duplicated genes. Blue represents duplicated genes with > 50% support rate. **d**–**f** Wordcloud plot of KEGG enrichment analysis of the union of expanded gene families and duplicated genes at Nodes 1, 3, and 8, respectively. Word size represents gene number in a pathway

Overall, 658 high-quality single-copy orthogroups were identified and used for phylogenetic tree construction (Table S11). Phylogenetic analysis indicated that among the analyzed Lamiaceae species, *C. americana* displayed the earliest divergence (73.11 Mya), whereas *T. mongolicus* and *T. quinquecostatus* diverged more recently (11.2 Mya) (Fig. 3a). Under this evolutionary framework, the nodes N1, N3, and N8 contained a significantly higher occurrence of gene duplication events (> 50% support rate) than other nodes (Fig. 3b and c, Table S12). Interestingly, the divergence times of these nodes corresponded to when the genome duplication events occurred based on the above genome synteny analysis.

Gene family analysis revealed that the nodes N1, N3, and N8 underwent expansion in 12, 809, and 1,271 gene families, respectively (Fig. 3a). GO and KEGG enrichment analyses were performed for these expanded gene families and the genes identified through gene duplication events. The results indicated significant enrichment of gene families at these nodes in GO terms of cellular process, metabolic process, and cellular anatomical entity (Fig. S5 and Tables S13–S15) and in KEGG pathways including plant hormone signal transduction, plant–pathogen interaction, and biosynthesis of secondary metabolites (Fig. 3d–f and Tables S16–S18). For *T. mongolicus* clade, the significantly expanded gene families (1,309) and duplicated gene orthogroups (4,182) (Fig. 3a and Table S19) were mainly enriched in GO terms of cellular process, metabolic process, and cellular anatomical entity and in KEGG pathways related to environmental adaptation and biosynthesis of secondary metabolites (Tables S20–S21).

### Genes related to the phenylpropanoid–flavonoid (PF) pathway

**Fig. 4:**
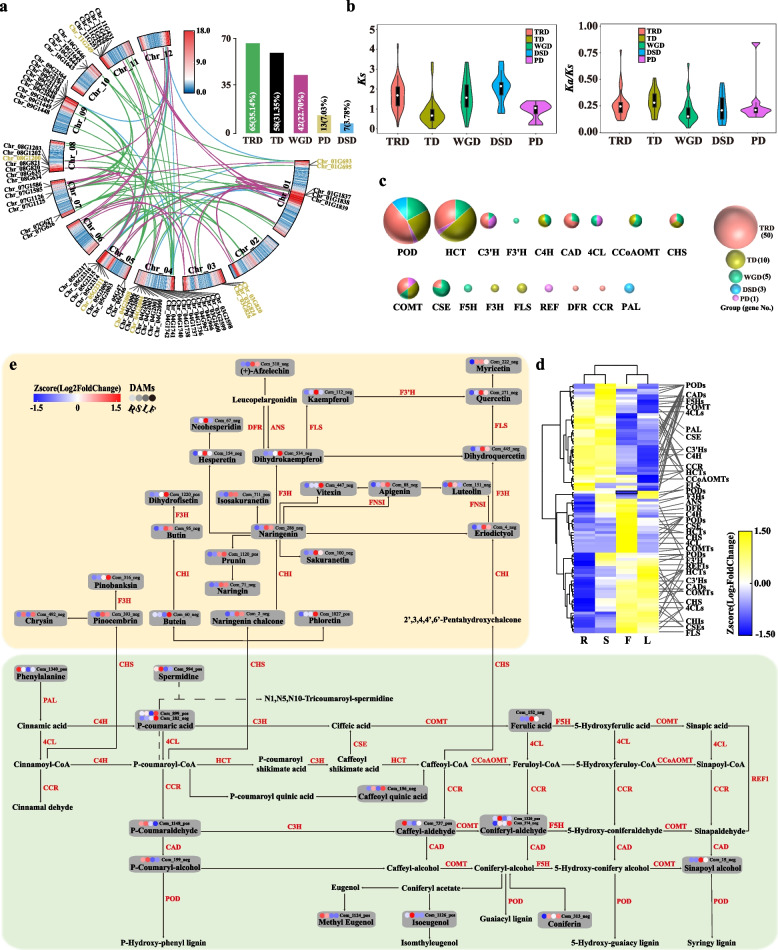
Gene composition, evolution, and expression characteristics of phenylpropanoid–flavonoid (PF) biosynthetic pathway in *T. mongolicus*. **a** Composition and location of PF-biosynthetic-pathway-related genes of *T. mongolicus*. The circle plot represents the distribution of genes associated with the PF pathway in the *T. mongolicus* genome. The bar chart in the upper right corner of the circle plot indicates the origin of the PF genes in the species. DSD, dispersed duplication; PD, proximal duplication; TD, tandem duplication; TRD, transposed duplication; WGD, whole-genome duplication. **b** *Ka*/*Ks* ratios and *Ks* values of gene pairs originating from different types of gene duplication. **c** Genes encoding key enzymes of PF pathway in *T. mongolicus* and their origin from different types of gene duplication. The color and size of the bubbles represent the duplication types and numbers of genes in the pathway. **d** Expression profiling of DEGs from PF pathway in four tissues in *T. mongolicus*. Abbreviations: *C4H*, *Cinnamate 4-monooxygenase*; *COMT*, *Caffeic acid 3-Omethyltransferase*; *CCR*, *Cinnamoyl-CoA reductase*; *4CL*, *4-coumarate-CoA ligase*; *HCT*, *Shikimate Ohydroxycinnamoyltransferase*; *REF1*, *Coniferyl-aldehyde dehydrogenase*; *CAD*, *Cinnamyl-alcohol dehydrogenase*; *CHI*, *chalcone isomerase*; *CHS*, *chalcone synthase*; *CCoAOMT*, *caffeoyl-CoA 3-O-methyltransferase*; *DFR*, *dihydroflflavonol 4-reductase*; *F3H*, *naringenin 3-dioxygenase*; *F5H*, *ferulate 5-hydroxylase*; *PAL*, *phenylalanine ammonia-lyase*; *POD*, *peroxidase*; *C3ʹH*, *5-O-(4-coumaroyl)-D-quinate 3*ʹ*-monooxygenase*; *F3ʹH*, *flavonoid 3'-monooxygenase*; *FLS*, *flavonol synthase*; *CSE*, *caffeoylshikimate esterase*; *ANS*, *leucoanthocyanidin dioxygenase*. **e** Metabolome characteristics of PF pathway in *T. mongolicus*. The colored circles represent the abundance of the metabolites identified in the PF pathway in four tissues

Overall, 211 genes belonging to 21 gene families were identified in the PF pathway. Among these genes, 174 were duplicated genes that could be classified into five categories. These genes were distributed on the gene-dense regions of *T. mongolicus* chromosomes (Fig. 4a) and were largely attributed to TRD (65, 34.57%), TD (58, 30.85%), and WGD (42, 22.34%). *Ks* and *Ka*/*Ks* values among different groups of duplicated genes revealed that TD gene pairs exhibited the smallest *Ks* value and highest *Ka*/*Ks* ratio (Fig. 4b).

Of the PF-pathway-related genes, 83 were DEGs, including *PALs*, *C4Hs*, and *4CLs*, across four tissues of *T. mongolicus* forming tissue-specific PF metabolic pathways (Fig. 4d). In the pathway, *POD* and *HCT* were the largest gene families (Fig. 4c and Table S22). Among the *PODs*, Chr_05G105 and Chr_05G2999 exhibited the highest expressed levels in the F tissue, with FPKM values of 100.03 and 76.94, respectively. In the S tissue, Chr_01G2755, Chr_01G3941, Chr_06G1469, and Chr_11G252 were relatively highly expressed, with expressions 2.05–58.74 times higher than those in other tissues. In the R tissue, Chr_06G2170 (FPKM 101.68) was the most abundant *POD*. Chr_04G545 was the most highly expressed *POD* in all tissues, with the highest expression in S (973.62), followed by F (573.12), L (508.51), and R (213.42) tissues. For *HCTs*, Chr_05G1038 exhibited high expression in F (82.28) and L (180.53) tissues, with 7.2- to 354-times higher expression than that in R (0.51) and S (11.37). Chr_08G1435 exhibited the opposite pattern, with FPKM of 343.77, 187.64, 15.01, and 3.16 in R, S, F, and L. respectively. For other genes, particularly those from the flavonoid pathway, higher expressions in F and L were identified, including one *F3H* (Chr_09G2363), one *CHS* (Chr_07G1122), one *CHI* (Chr_01G4712), one *ANS* (Chr_08G1514), and one *F3ʹH* (Chr_04G1799).

### Metabolites in the PF pathway

In the PF pathway, 48 metabolites were identified, and the abundance of these metabolites exhibited a general trend that it was higher in the aboveground tissues (F, L, and S) than in the belowground tissues (R), particularly for the metabolites of flavonoid pathway (Fig. 4e and Table S23). Of the metabolites, 22 were differential metabolites (DAMs) among the four tissues, and 15 of them exhibited relatively high abundance in the metabolites datasets (Table S24). Specifically, L-phenylalanine was highly abundant in F (content 2- to 8.3-folds higher than that in L, R, and S). The contents of apigenin, naringenin chalcone, butin, and naringenin were relatively high in F and L, with abundance up to 3 times higher than that in R. The abundance of coniferin was higher in L and S.

Besides, as intermediates of the PF biosynthesis pathway, several high-abundance metabolites were detected in the *T. mongolicus* metabolome (Fig. 4e). These metabolites included various apigenin-related compounds, including apigenin 7-O-beta-D-glucuronide, apigenin 5-O-glucoside, and apigenin O-hexosyl-O-pentoside (Table S23). These metabolites were particularly abundant in F and L, with apigenin 7-O-beta-D-glucuronide exhibiting the highest abundance in the entire metabolome of *T. mongolicus*.

### Correlation of gene expression and metabolite synthesis in PF pathway

**Fig. 5:**
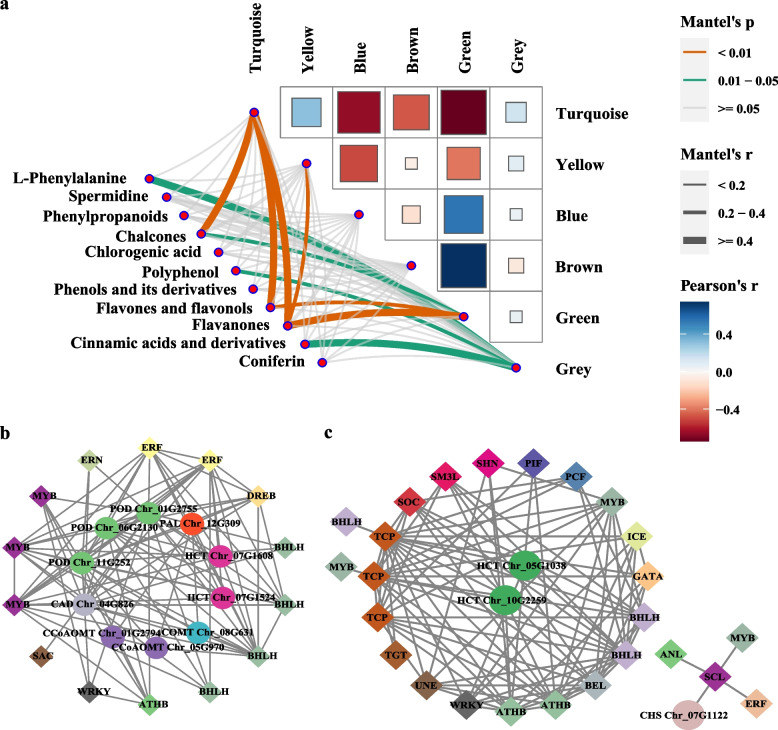
Gene co-expression network and transcription factor (TF) identification. **a** Weighted gene co-expression network analysis of genes and metabolites identified in the PF pathway in *T. mongolicus*. **b** and **c** Transcriptional regulatory network of PF-pathway-related genes and TFs in green and yellow modules. Diamonds and circles represent PF-pathway-related genes and their regulated TFs, respectively. Lines indicate correlation

To identify whether gene expression regulated relevant metabolite synthesis, the correlation between the expression of PF-pathway-related genes and abundance of their downstream metabolites was investigated (Table S25). Green and yellow modules were significantly associated with the abundance of chalcones and dihydrochalcones, flavones and flavonols, and flavonoids (Fig. 5a; *P* < 0.05). In the yellow module, three *PODs*, two *HCTs*, two *CCoAOMTs*, one *PAL*, one *CAD*, and one *COMT* were identified (Fig. 5b). In the green module, two *HCTs* and one *CHS* were identified (Fig. 5c). Besides, TFs annotated in the *T. mongolicus* genome were included in the gene co-expression network analysis, and 39 TFs possibly playing significant roles in regulating the expression of genes in the two modules were identified (Fig. 5b and c, S6). These TF families mainly include MYB, bHLH, TCP, ERF, and ATHB, which are well-known for their crucial roles in plant growth and development, stress resistance, and secondary metabolite synthesis.

## Discussion

*Thyme* plants and their essential oil components possess antibacterial, antiviral, and antioxidant properties. Therefore, they are widely used in the pharmaceutical, food, and cosmetic industries [85–87]. Revealing the composition and characteristics of the *T. mongolicus* genome is crucial for the development and utilization of its genetic resources. In this study, a high-quality chromosome-level genome of *T. mongolicus* was generated. K-mer analysis revealed that the genome size of *T. mongolicus* was 559.25 Mb, with heterozygosity and repetitive sequences accounting for 1.99% and 57.64%, respectively (Table S1), indicating a complex genome structure in this species. The high heterozygosity poses challenges for genome assembly [88]. In this study, 56.5 Gb HiFi-reads were assembled into a 605.2 Mb genome (Table 1 and Table S2). The assembled genome size exceeded the size estimated by K-mer analysis, possibly due to the impact of the high heterozygosity of *T. mongolicus* genome [89]. To obtain a chromosome-level *T. mongolicus* genome, 47.53 Gb Hi-C data were anchored to the 12 assembled chromosomes, the total length of which was 582.7 Mb, accounting for 96.28% of the whole assembled genome (Fig. 1a, Tables S2 and S3). BUSCO analysis revealed that the assembled *T. mongolicus* genome has a high level of completeness [46], including 93.74% single-copy and 3.84% duplicated complete genes (Fig. S2 and Table S4). The LTR Assembly Index (LAI) of the *T. mongolicus* genome exceeds 20 (Fig. 1b), reaching the gold standard and indicating good contiguity of the assembled *T. mongolicus* genome [47]. Collectively, the chromosome-level genome of *T. mongolicus* would enrich the phylogenetic information of the Lamiaceae plants and lay a foundation for the further studies on genome evolution and unique biological characteristic of *T. mongolicus*.

The duplication of genomes has a profound impact on plant evolution and is a significant driving factor in the formation of species, adaptability, and specific biological traits [90, 91]. In this study, the *T. mongolicus* genome underwent two rounds of genome duplication, i.e., an ancient triplication and a subsequent duplication event (Fig. 2). The genome evolution of *V. vinifera* appears relatively conserved, and its ancient triplication event (γ) has been well-studied and generally used as a reference in the study of genome evolution in eudicots [92]. The genomic syntenic relationship between *T. mongolicus* and *V. vinifera* primarily follows 1:3 pattern, consistent with the ancient triplication characteristic of *V. vinifera*. However, the depth of syntenic blocks between *V. vinifera* and *T. mongolicus* ranges from 1 to 6, mostly in 1:2 relationship, supporting the occurrence of polyploidization in the *T. mongolicus* genome. This is similar to the findings from the comparison of grape and cucumber genomes [68]. Furthermore, the *T. mongolicus* genome has experienced frequent chromosomal rearrangements and other structural variations following genome duplication (Fig. 2a), tending toward a diploidized genome. As indicated in Fig. 2b, the genomes of *T. mongolicus* and other species within the Lamiaceae family (*S. bowleyana*, *S. hispanica*, and *T. quinquecostatus*) retain traces of ancient triplication; however, the corresponding syntenic blocks exhibit a relatively inconspicuous *Ks* peak (*Ks* = 1.87–2.07) (Fig. 2b). Interestingly, the genome triplication of *C. americana* is well-preserved and occurred at approximately 116.14 Mya (*Ks* ≈ 2), consistent with previous reports [93]. It is consistent with the shared γ genome duplication event in angiosperms (Fig. 2c) [94], thus indirectly supporting the ancient triplication events in Lamiaceae species.

The majority of angiosperms have experienced genome duplication at the K-P boundary, which has had significant implications for angiosperm evolution, serving as both a mechanism for adapting to dramatic environmental changes and the foundation of the current diversity of angiosperms [94]. In this study, except for *C. americana*, the genomic syntenic regions of *T. mongolicus*, *T. quinquecostatus*, *S. bowleyana*, and *S. hispanica* exhibit peaks in *Ks* values ranging from 1.04 to 1.21 (Fig. 2b), indicating genome duplications occurring approximately 60.39 to 70.27 Mya; this is consistent with WGD events reported for *T. quinquecostatus* and *S. bowleyana* [71, 95]. With the differentiation of Lamiaceae plants and environmental selection, the genomic evolution of Lamiaceae species demonstrates certain diversity. *C. americana* and *S. hispanica* underwent additional WGD events approximately 19.74 and 29.62 Mya, respectively (*Ks* = 0.34 and 0.51) (Fig. 2b).

Numerous studies have highlighted the significant role of repetitive sequences in the process of genome evolution [96, 97]. Plants with relatively larger genomes such as *Ginkgo biloba*, *Camellia sinensis*, and *Taxus chinensis* exhibit slow, stable, and long-term insertion of LTR retrotransposons [98–100]. The Ty3/Gypsy families play a crucial role in the expansion of the *Vernicia fordii* genome [101]. In the coral genome, extensive tandem repeats drive the expansion of gene families [102]. In *T. mongolicus*, repetitive sequences constitute over 70% of the genome and are nonrandomly distributed along chromosomes and overlapped with gene density regions (Fig. 1a). This reflects the relationship between gene duplication and the insertion of repetitive sequences. Among LTR retrotransposons, the Ty3/gypsy and Ty1/copia elements were the most prevalent, especially for the Reina and AIe subfamilies, likely playing a primary role in driving the gene duplication in this species. LTR bursts may persist in the genomic evolution of certain plant taxa [99]. Analysis of the insertion time and quantity of LTR retrotransposons revealed that their insertion in the genomes of *T. mongolicus*, *T. quinquecostatus*, and *S. bowleyana* was a continuous process and had significantly increased in the most recent 1 Mya (Fig. 2d). This may further impel the divergence of these species.

Besides, in a previous study of *T. quinquecostatus*, collinear regions with a *Ks* peak of 0.07 were considered a WGD event [71]. In this study, we found similar *Ks* curves of syntenic genes between *T. mongolicus* and *T. quinquecostatus* genomes, suggesting a close phylogenetic relationship and similar genome evolution histories of these two species. Investigation of the chromosomal positions of genes distributed in these syntenic regions revealed that they were mostly located in adjacent positions on relevant chromosomes. Therefore, we proposed that these homologous genes were more likely to be generated by tandem repeats.

Phylogenetic analysis is widely employed to explore evolutionary history and relationships of species. In this study, a phylogenetic tree was constructed using 658 single-copy genes, and the phylogenetic relationships among the analyzed Lamiaceae plants was clarified. It revealed that after the divergence of monocots and dicots (160.44 Mya), Lamiaceae species diverged from the lineages of *V. vinifera* and *A. thaliana* by approximately 118.36 Mya (Fig. 3a). This was consistent with the report on *Scutellaria barbata* [103] and was in line with the results of our *Ks* analysis based on genome collinearity. Furthermore, it identified that *C. americana* diverged earlier from the Lamiaceae plants (73.11 Mya), while the formation of *T. mongolicus* and *T. quinquecostatus* occurred relatively recently, with a divergence time of 11.2 Mya (Fig. 3a).

Based on highly confident evolutionary relationships, researchers can gain information on the evolutionary history and direction of plant genomes. Core eudicots represented by *Akebia trifoliata* experienced two WGD events during the Jurassic–Cretaceous boundary (146.63 Mya) and the mid-Cretaceous period (85.15 Mya). This contributed to the retained duplicate genes related to adaptation to atmospheric CO_2_ concentration, drought, and habitat salinity changes and the evolution of various strategies to adapt to stress [104]. In *T. mongolicus*, the significantly enriched expanded and duplicated gene families were enriched in plant hormone signal transduction, MAPK signaling pathway, and plant–pathogen interactions. This suggested that the genomes of Lamiaceae species retain genes associated with environmental adaptation, providing them with the potential to survive under different stress conditions. For example, plant-hormone-related genes play important roles in disease resistance and defense in potatoes [105]; genes related to plant hormone signal transduction were significant for salt tolerance in *Helianthus tuberosus* [106], and the retention and evolution of the MAPK signaling pathway was crucial for plants to respond to drought, low temperature, high salinity, nutrient deficiency, and other abiotic stresses [107]. The plant–pathogen interaction pathway can regulate plant-specific adaptations by increasing or decreasing plant sensitivity to abiotic and biotic stresses [108].

Evolution occurs at different times and spatial scales after genome duplication, promoting the formation of specific species and biological traits. An interesting case is the study of carnivorous plants, including *Dionaea muscipula*, *Aldrovanda vesiculosa*, and *Drosera spatulata*, which revealed significant expansions of gene families related to prey attraction and perception, digestion, nutrient absorption, and transport. However, the genes associated with root development were significantly lost in *D. spatulata* [109]. This genomic evolutionary feature is particularly evident in the study of medicinal plants. For instance, in the genome of *Angelica sinensis*, genes involved in the biosynthesis of furanocoumarins have independently evolved in the families Moraceae, Fabaceae, Rutaceae, and Apiaceae after the ζ and ε WGD [110]. *Arctium lappa* retains specific genes that involved in cellulose metabolism, which may play an important role in the formation of cell walls in this plant [111]. *Taxus chinensis* contains gene clusters formed by gene duplication that are responsible for the biosynthesis of taxane, contributing to the production of taxol [100]. In the present study, *Thyme* plants retained a large number of genes associated with environmental adaptation and secondary metabolite biosynthesis. These genes were annotated to pathways such as photosynthesis, plant hormone signal transduction, flavonoid biosynthesis, and diterpenoid and triterpenoid biosyntheses, which may be closely related to the habitat adaptation and aromatic characteristic of *T. mongolicus*.

Phenylpropane–flavonoid (PF) metabolic pathway is the most important secondary metabolic pathway in plants. Combined with genomic evolution, transcriptome, and metabolome analysis, we proposed that this metabolic pathway not only plays an important role in the habitat adaptation of *T. mongolicus* but also provides material basis for the formation of its aromatic property.

In terms of genomic evolution, genes associated with the PF pathway in *T. mongolicus* were primarily located within gene-dense regions of the chromosomes. The majority of these genes had their origins in TRD, TD, and WGD. Some of the TD events led to the subsequent development of extensive gene families, such as *POD* and *HCT* gene families. The relatively higher *Ks* values of WGD and TRD-related genes suggested that this pathway may have been continuously selected during the evolution of the *T. mongolicus* genome. In contrast, genes resulting from more recent TD events exhibited lower *Ks* values, indicating their status as recent evolutionary hotspots in this species. This highlights the widespread increase in gene copy numbers driven by WGD and TRD, which laid the foundation for the speciation of *T. mongolicus*. Furthermore, under specific environmental selection, some genes became intrinsic factors contributing to the species’ distinctiveness. *T. mongolicus* predominantly thrives in arid, high-temperature, and barren soil environments in northwestern China and is often subjected to wound and pathogenic infections caused by grazing. These environmental factors are reported to induce increased activity of enzymes related to this pathway, consequently promoting the accumulation of specific metabolites [112–114]. Subsequently, these metabolites function as environmental response factors or signaling molecules, playing roles in growth, adaptation to stress, and the accumulation of specialized metabolites [115–117].

When examining gene expression in the PF pathway of *T. mongolicus*, a total of 174 coding genes encoding 20 enzymes were identified. Among them, 67 were differentially expressed across various tissues, including roots, stems, leaves, and flowers, forming a functionally complementary PF metabolic pathway (Fig. 4d). PAL, C4H, and 4CL are key enzymes in the phenylpropane metabolic pathway. PAL catalyzes the conversion of phenylalanine into cinnamic acid (CA), followed by the transformation of CA into P-coumaric acid by C4H. 4CL, in turn, catalyzes the synthesis of coenzyme A esters, including CA and P-coumaric acid, further branching into the lignin and flavonoid biosynthesis pathways. Numerous studies have demonstrated that *PAL* genes can respond to various environmental stimuli, including pathogen infections, mechanical damage, nutrient deficiencies, UV radiation, extreme temperatures, and so on. In *Senna tora*, different *PAL* gene members had promoter regions rich in hormone and stress response elements, as well as growth and development regulatory elements [118]. These genes exhibited significant differences in expression levels across various tissues in the species. *pal1* and *pal2* double mutants of *A. thaliana* exhibited particular sensitivity to UV-B radiation but greater tolerance to drought conditions [119]. The *T. mongolicus PAL* genes Chr_12G309 and Chr_01G3921 were highly expressed in the root and stem, suggesting their potentially significant roles in initiating the phenylalanine metabolism pathway in different tissues of the plant. P-coumaric acid is a component of plant cell walls, serving as a major phenolic acid that can restrict cell wall degradation, thus playing a crucial role in maintaining plant growth and morphology [120]. In this study, the *C4H* gene Chr_01G1839 exhibited a high expression level in the flower, reaching up to 710.96, which was likely closely related to the high abundance of 4-CA in the flowers and its critical role in lignin synthesis in *T. mongolicus*. Furthermore, the relatively high expression level of the *4CL* gene Chr_12G1703 in F and L suggested its importance in maintaining *T. mongolicus* growth in drought environments. This finding aligns with the observations in *Gossypium hirsutum*, where the silencing of *Gh4CL7* led to increased sensitivity to drought stress and *Gh4CL7* overexpression enhanced the drought tolerance in transgenic *A. thaliana* [121].

POD is a well-studied enzyme class in plants closely associated with defense against biotic and abiotic stressors. For instance, *AtPOD3* has been reported to positively regulate salt and drought stress responses in *A. thaliana* [122]. Overexpression of *AtPOD39* and *AtPOD64* enhances the tolerance of *A. thaliana* and tobacco to low temperatures and aluminum stress, respectively [123]. Overexpressing wheat *TaPOD* can restore the salt sensitivity in *A. thaliana*. Under drought stress, the antioxidant defense system in *T. mongolicus* was activated. It effectively cleared reactive oxygen species (ROS) in the plant and alleviated membrane lipid peroxidation damage, representing a drought response strategy [124]. Our study reported that the *POD* genes Chr_11G252, Chr_05G105, and Chr_01G2755 exhibited high homology (E-value < 1.0E-50) with *AtPOD3*, *AtPOD39*, and *AtPOD64*, respectively. Therefore, it is speculated that these genes in *T. mongolicus* have similar functions in clearing ROS during its adaptation to drought environment.

HCT is an acyltransferase that utilizes p-coumaroyl-CoA as a substrate to channel phenylpropanoid metabolism into lignin monomer synthesis [125]. In *Medicago sativa*, downregulation of *HCT* alters lignin content and composition and increases flavonoid levels in the roots and stems, enhancing stress tolerance [126]. In *A. thaliana*, the *HCT* mutant line exhibits growth defects attributed to elevated levels of flavonoid compounds [127]. In *T. mongolicus*, significant differences in *HCT* gene expression were observed. Chr_08G1435 exhibited almost tissue-specific and high expression in the root and stem, whereas Chr_05G1038 was highly expressed in the flower and leaves. This reflected their complementary roles in different tissues and served as potential targets for research on lignin and flavonoid synthesis regulation in this plant.

At the metabolomic level, 48 metabolites were detected in the PF pathway of *T. mongolicus*, including 12 flavones, 11 flavonols, 7 flavonones and flavanols, 5 phenolic acids, 3 chalcones and dihydrochalcones, and 10 other substances. Among these, compounds such as flavanols, flavonoids, and anthocyanins are well-known essential substances involved in plant stress response and defense. This again reflects the critical role of this pathway in the environmental adaptation of *T. mongolicus*. However, interestingly, in the PF pathway of *T. mongolicus*, the majority of the identified metabolites, especially those related to the flavonoid pathway, exhibited higher abundance in the flowers and leaves. This suggested its additional function in the species.

In flowering plants, volatile organic compounds are primarily released from the floral organs, and in some herbaceous aromatic plants such as mint and basil, they are released from the leaves [128–130]. Studies have reported that *Thymus* species possess various volatile components in their flowers and leaves, and they contribute to a significant part of their aromatic properties. In the leaves of *T. zygis*, phenolic compounds such as rosmarinic acid, caffeic acid, ferulic acid, and guanosine were identified, along with flavonoid compounds including quercetin, apigenin, and luteolin [131]. Fresh leaves of *T. vulgaris* are characterized by the presence of phenolic compounds including rosmarinic acid, caffeic acid, luteolin, and p-coumaric acid [132]. In *T. mongolicus*, the highest content of volatile substances was observed in the leaves, and volatile components in the leaves, lateral branches, main branches, and roots exhibited distinct tissue specificity [133].

Plants produce a diverse array of volatile organic compounds, which can be categorized into three major classes based on their structures: terpenoids, phenylpropanoids/benzenoids, and fatty acids [134]. Phenylpropanoids/benzenoids are essential components responsible for generating fragrance in plants [135]. Phenylalanine, as the initial substrate of the PF pathways, undergoes various enzymatic reactions to enter the phenylpropane and flavonoid metabolic branches. In the metabolome of *T. mongolicus*, phenylalanine was found in high abundance, indicating the high demand for the PF pathway in the species.

Naringenin chalcone, a precursor of flavonoid synthesis, is catalyzed by CHS, which is a key enzyme in the flavonoid metabolic pathway. Subsequently, another crucial enzyme, CHI, catalyzes the isomerization of chalcone to form naringenin. In *T. mongolicus*, both the *CHS* gene (Chr_07G1122) and the *CHI* gene (Chr_01G4712) were highly expressed in F and L, consistent with the high abundance of their respective metabolites. This phenomenon suggested that the catalysis of these enzymes effectively increases the flux from the phenylalanine metabolic pathway to the flavonoid metabolic pathway, forming the main route of the PF metabolic pathway in *T. mongolicus*. This was consistent with the close relationship observed in other plants such as *Mentha canadensis* and *Nematanthus glabra* between the synthesis of volatile organic compounds and regulation of corresponding gene expression [136, 137].

Flavonol compounds and their glycosylated forms, catalyzed by flavonol synthase (FLS), are also potential aroma constituents in plants [138]. In this study, a total of 12 flavonol compounds were identified, including quercetin, isorhamnetin, naringenin, kaempferol, naringin, apigenin, dihydroquercetin, and luteolin. Among them, quercetin, isorhamnetin, and naringenin were found in high abundance, suggesting that they serve as important precursors for the formation of aromatic compounds in the flowers and leaves of *T. mongolicus*. For example, compounds such as naringenin, hesperetin, isosakuranetin, and eriodictyol form flavanone-7-O-glucosides under the catalysis of 7-O-glucosyltransferase (7-GlcT). Apigenin is converted into rhoifolin under the catalysis of 7-O-glucosyltransferase (7-GlcT) and 1,2-rhamnosyltransferase (1,2-RhaT) [139]. Apigenin reacts with glucuronic acid lactone to form apigenin-7-O-β-D-glucuronide [140].

TFs play a crucial regulatory role in the synthesis of specific compounds by regulating the expression of genes related to PF pathway [141]. Co-expression network analysis indicated that TFs such as MYB, AP2/ERF, WRKY, and bHLH interact intricately with genes including PAL, PODs, CADs, CCoAOMT, 4CL, and F3ʹH. This finding enhanced the understanding of the regulatory network of the PF pathway in *T. mongolicus* and laid a foundation for further elucidating the molecular mechanisms of the pathway functioning in the environmental adaptation and aromatic compound synthesis in this species.

## Conclusion

In this study, a chromosome-level reference genome of *T. mongolicus* was constructed. Genome evolution analysis revealed that *T. mongolicus* underwent two WGD events. One was an ancient triplication events, and another was a recent event occurring at approximately 70.27 Mya. Insertion of LTR retrotransposons significantly increased in the most recent 1 Mya, possibly due to the recent tandem repeats. Comparative genomic analyses revealed an estimated divergence time of *T. mongolicus* from its close relative *T. quinquecostatus* at approximately 11.2 Mya. Functional enrichment analysis of expanded gene families and duplicate genes at evolutionary nodes revealed that these genes are associated with environmental adaptability and biosynthesis of secondary metabolites. Integrating transcriptome and metabolomic analyses, the key genes and metabolites involved in the PF metabolic pathway were identified, laying the foundation for further research on the molecular mechanisms underlying the accumulation of related substances in this plant and the extraction of specific compounds.

### Supplementary Information

**Supplementary Material 1.**

**Supplementary Material 2.**

## Data Availability

The sequenced Hifi, Hi-C, and transcriptome data have been deposited in the SRA database with the accession number SUB13972845.

## Abbreviations

Hi-C: High throughput chromosome conformation capture
BLAST: Basic Local Alignment Search Tool
MAFFT: Multiple Alignment using Fast Fourier Transform
BUSCO: Benchmarking Universal Single-Copy Orthologs
CDS: Coding sequence
Gb: Giga base pairs
Mb: Mega base pairs
bp: Base pairs
GC: Guanine-cytosine
kb: Kilobase pairs
GO: Gene Ontology
KEGG: Kyoto Encyclopedia of Genes and Genomes
LINE: Long interspersed nuclear element
LTR: Long terminal repeat
PacBio: Pacific Biosciences
PAML: Phylogenetic Analysis by Maximum Likelihood
RNA-seq: RNA sequencing
rRNA: Ribosomal RNA
miRNA: MicroRNA
snRNA: Small nuclear RNA
tRNA: Transfer RNA
SINE: Short interspersed nuclear element
TE: Transposable element
TF: Transcription factor
WGD: Whole genome duplication
Ks: Pairwise synonymous substitution rates
