# Supplementary material for: Chromosome-level genome assembly provides insights into the genome evolution and functional importance of the phenylpropanoid–flavonoid pathway in Thymus mongolicus
Source: BMC Genomics. 2024 Mar 19;25:291. doi: 10.1186/s12864-024-10202-8 (PMC10949689; doi:10.1186/s12864-024-10202-8)
Supplement: Supplementary file 2 — Supplementary Material 2. [file 12864_2024_10202_MOESM2_ESM.pdf]

Supplementary Figures

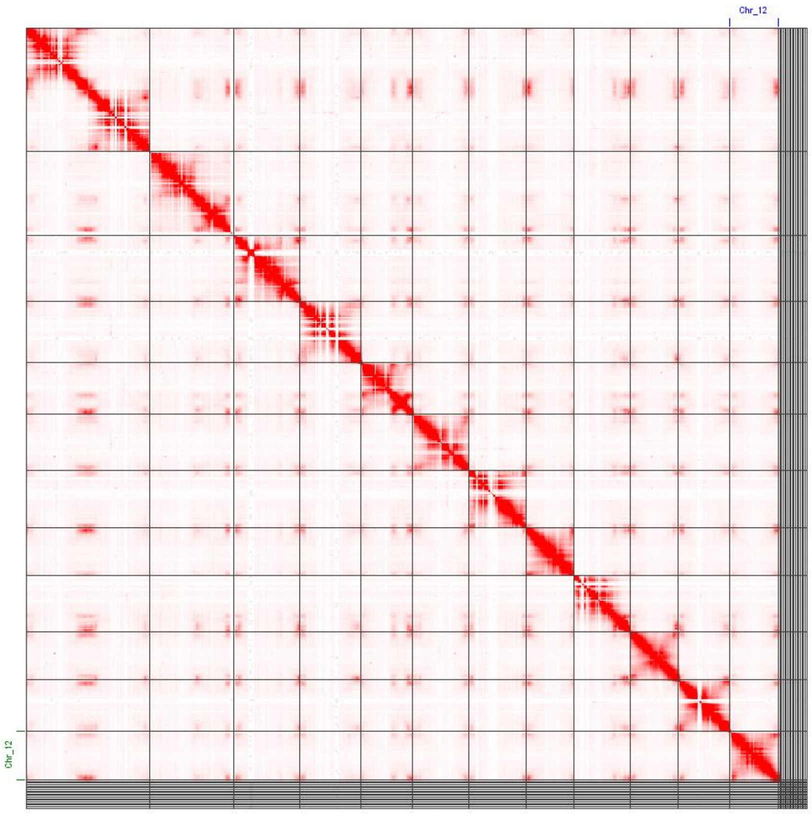

Supplementary Figure 1. Chromosome-level assembly of the *T. mongolicus* genome using Hi-C technology.

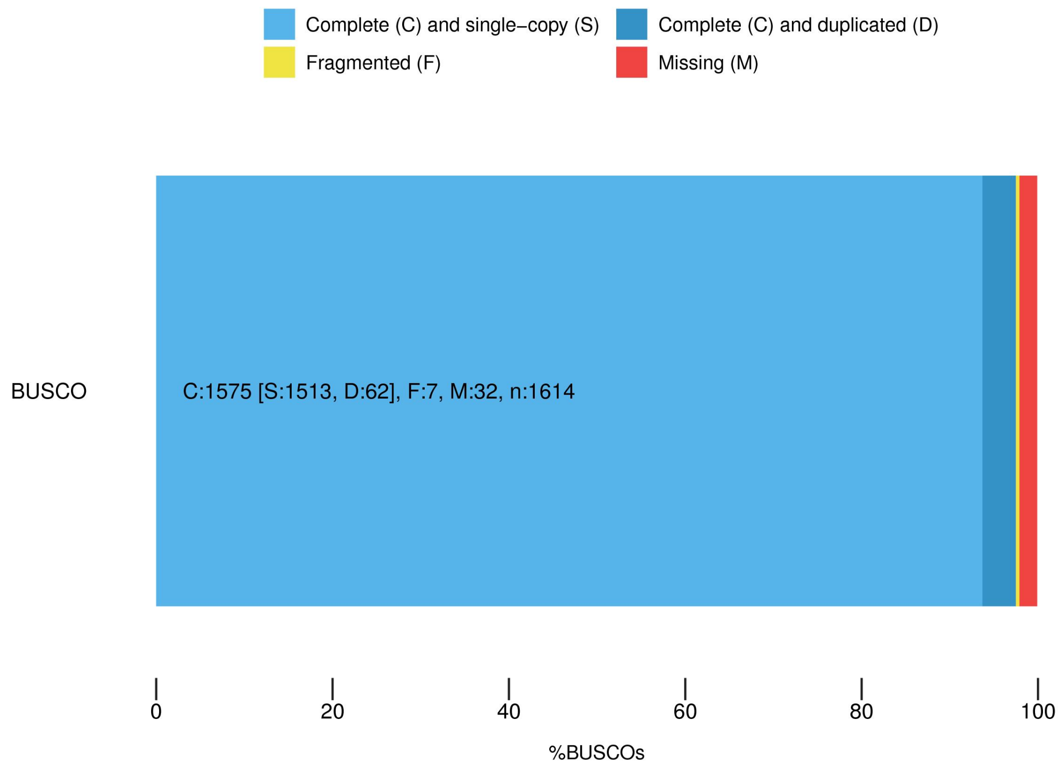

Supplementary Figure 2. BUSCO results for the *T. mongolicus* genome assembly.

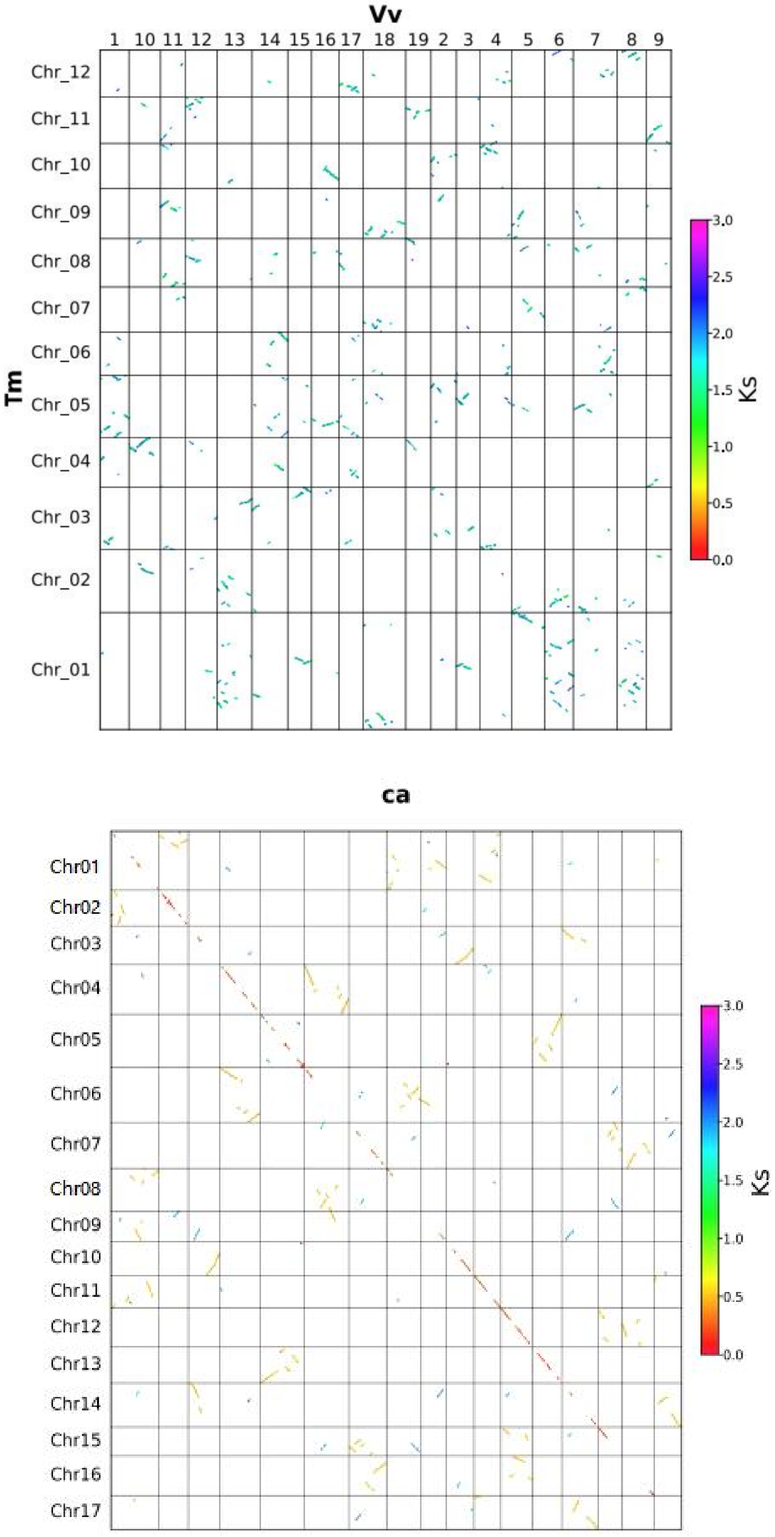

A

B

Supplementary Figure 3. The intergenomic collinearity relationship of the two genomes. (A) The dot plot of synteny blocks between *T. mongolicus*-*Vitis vinifera.* (B) Intra-genomic comparison within *C. americana*.

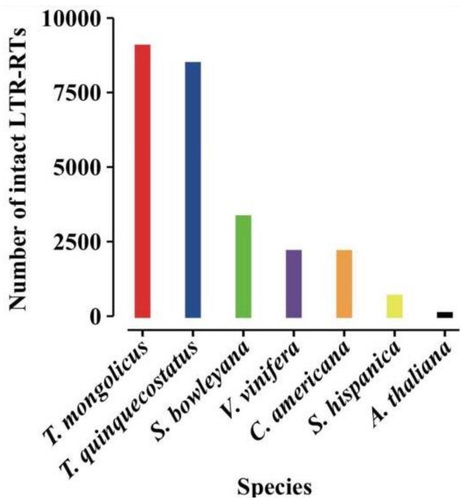

Supplementary Figure 4. The number of intact LTR-RTs in *T. mongolicus* and other species.

A

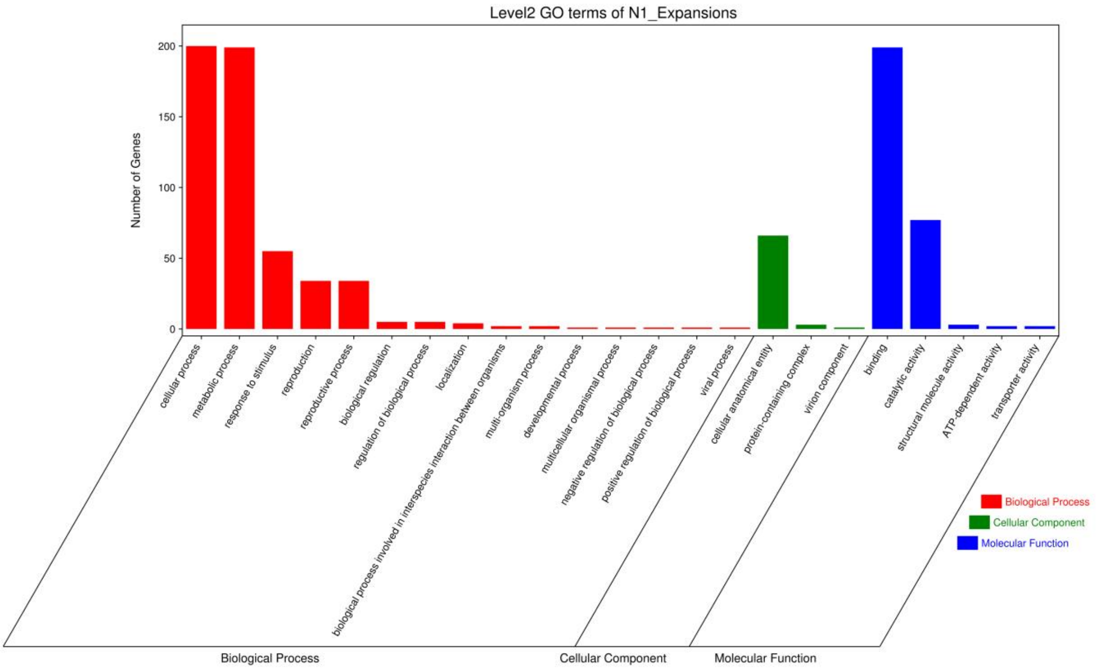

B

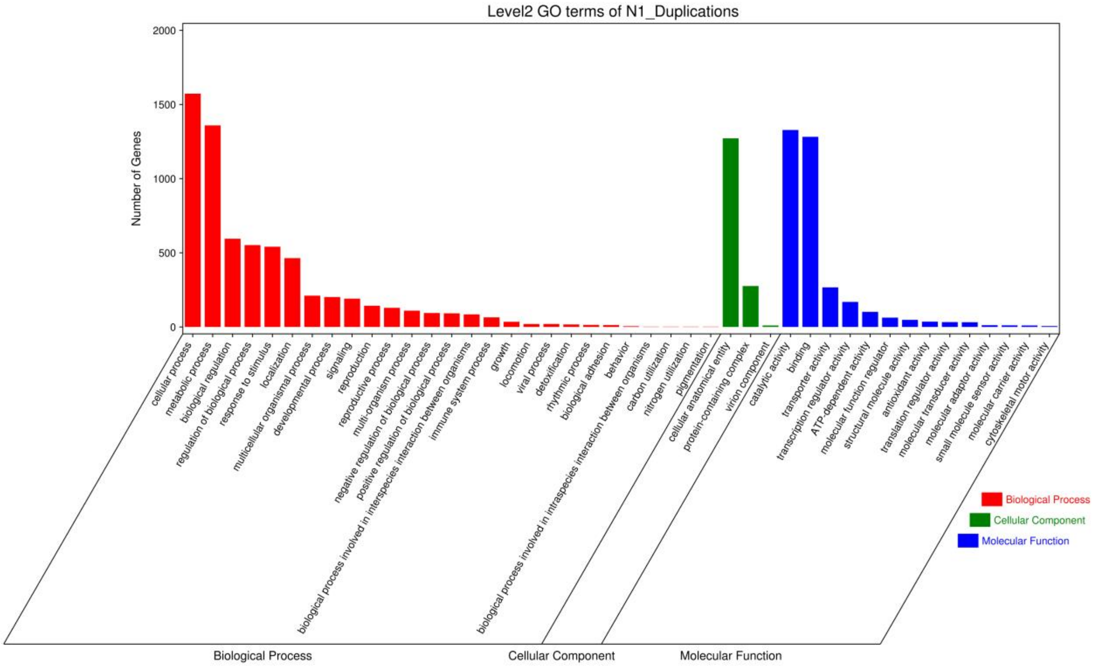

Level2 GO terms of N1_Duplications
Number of Genes
2000
1500
1000
500
0
cellular process
metabolic process
biological regulation
regulation of biological process
response to stimulus
localization
multicellular organismal process
developmental process
signaling
reproduction
reproductive process
multi-organism process
negative regulation of biological process
positive regulation of biological process
biological process involved in interspecies interaction between organisms
immune system process
growth
locomotion
viral process
detoxification
rhythmic process
biological adhesion
behavior
biological process involved in intraspecies interaction between organisms
carbon utilization
nitrogen utilization
pigmentation
cellular anatomical entity
protein-containing complex
virion component
catalytic activity
binding
transporter activity
transcription regulator activity
ATP-dependent activity
molecular function regulator
structural molecule activity
antioxidant activity
translation regulator activity
molecular transducer activity
molecular adaptor activity
small molecule sensor activity
molecular carrier activity
cytoskeletal motor activity
Biological Process
Cellular Component
Molecular Function
Biological Process
Cellular Component
Molecular Function

C

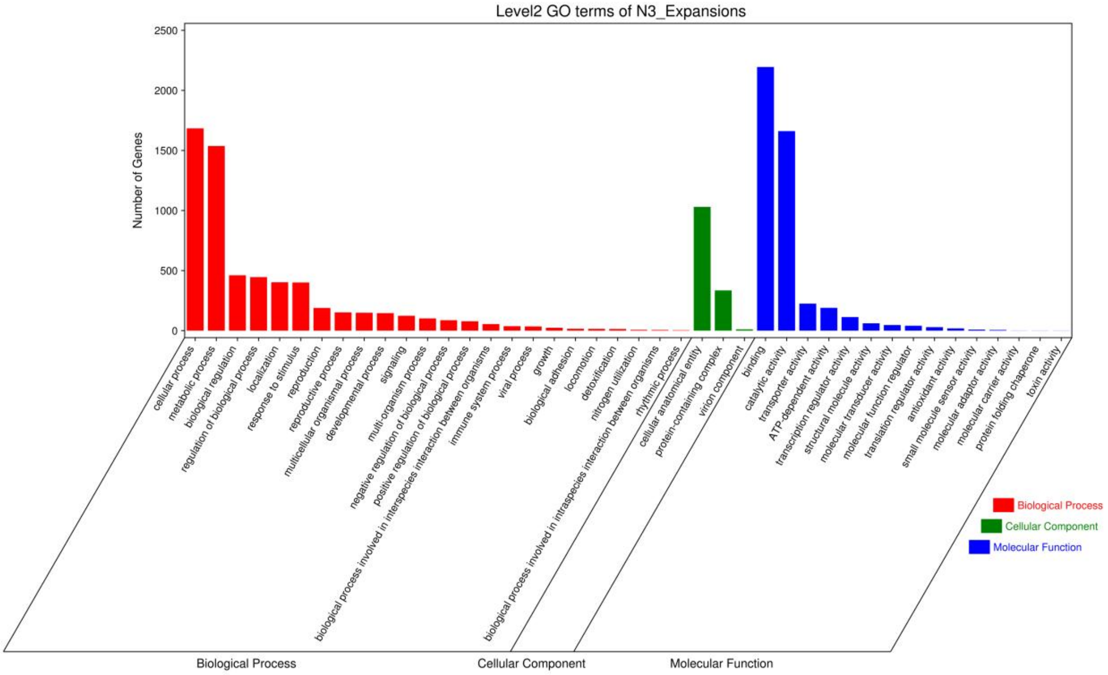

Level2 GO terms of N3_Expansions
Number of Genes
2500
2000
1500
1000
500
0
cellular process
metabolic process
biological regulation
regulation of biological process
localization
response to stimulus
reproduction
reproductive process
multicellular organismal process
developmental process
signaling
multi-organism process
negative regulation of biological process
positive regulation of biological process
biological process involved in interspecies interaction between organisms
immune system process
viral process
growth
biological adhesion
locomotion
detoxification
nitrogen utilization
biological process involved in intraspecies interaction between organisms
rhythmic process
cellular anatomical entity
protein-containing complex
virion component
binding
catalytic activity
transporter activity
ATP-dependent activity
transcription regulator activity
structural molecule activity
molecular transducer activity
molecular function regulator
translation regulator activity
antioxidant activity
small molecule sensor activity
molecular adaptor activity
molecular carrier activity
protein folding chaperone
toxin activity
Biological Process
Cellular Component
Molecular Function
Biological Process
Cellular Component
Molecular Function

D

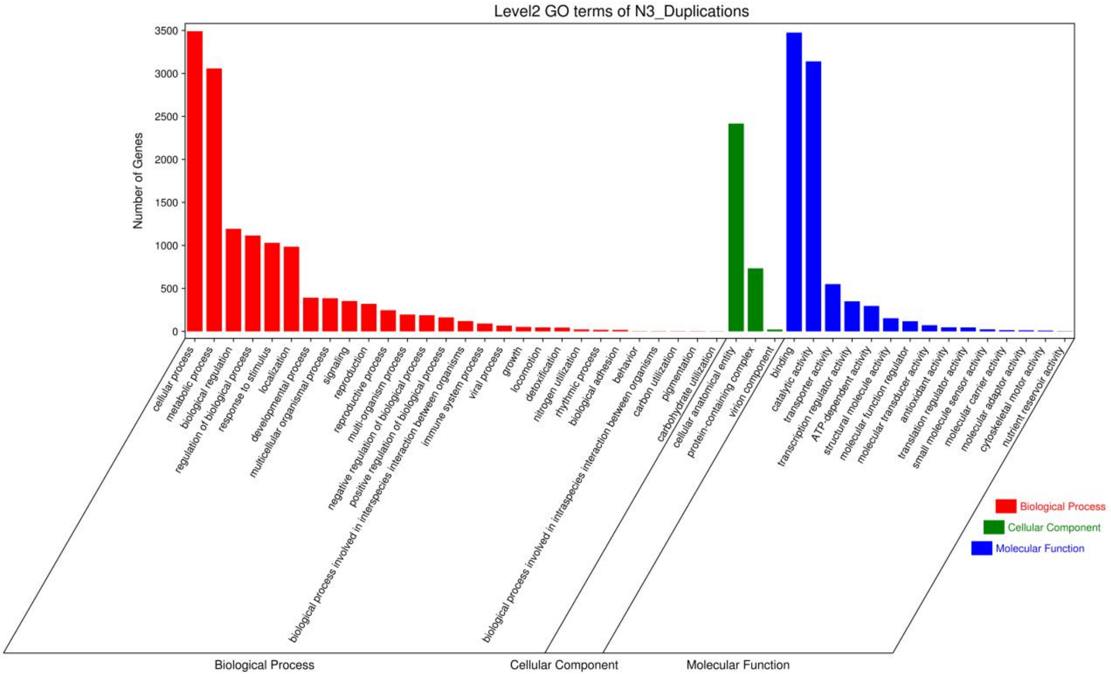
Level2 GO terms of N3_Duplications
Number of Genes
Biological Process
Cellular Component
Molecular Function

E

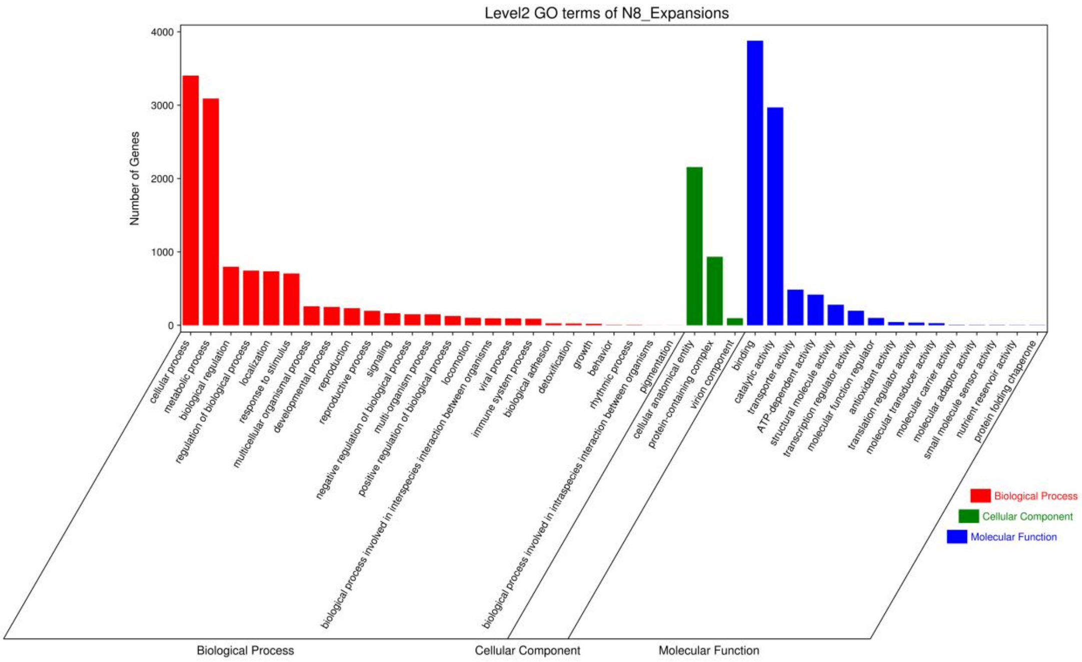
Level2 GO terms of N8_Expansions
Number of Genes
Biological Process
Cellular Component
Molecular Function

F

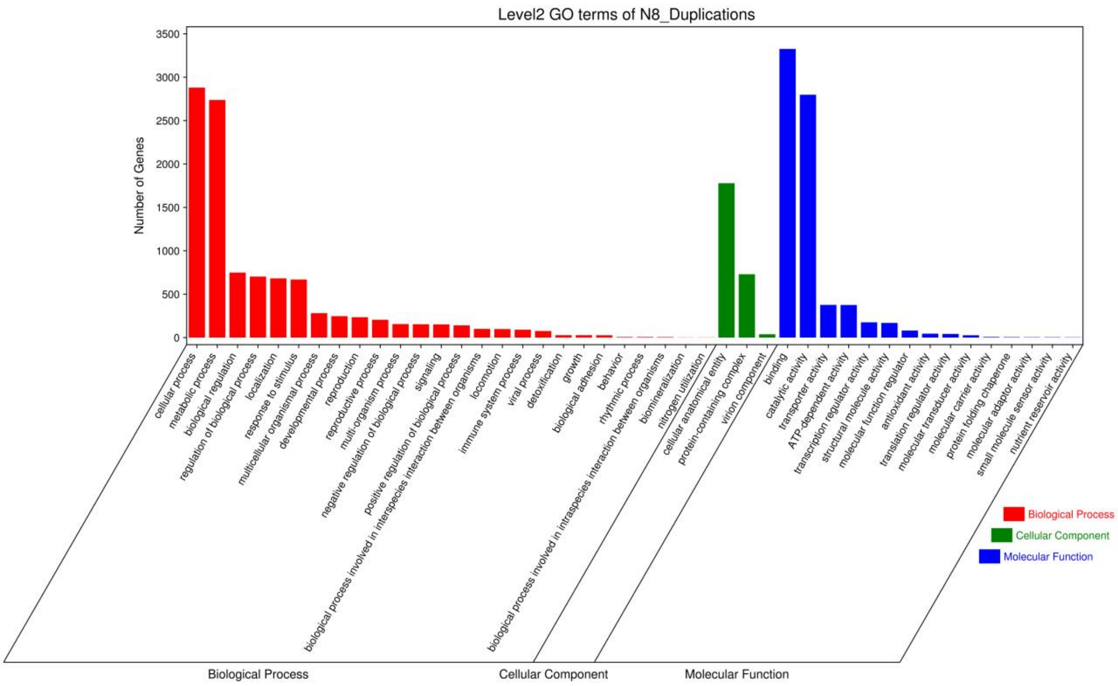

Supplementary Figure 5. GO enrichment analysis of genes in three nodes. (A) GO enrichment analysis of expanded genes in node N1. (B) GO enrichment analysis of duplicated genes in node N1. (C) GO enrichment analysis of expanded genes in node N3. (D) GO enrichment analysis of duplicated genes in node N3. (E) GO enrichment analysis of expanded genes in node N8. (F) GO enrichment analysis of duplicated genes in node N8.

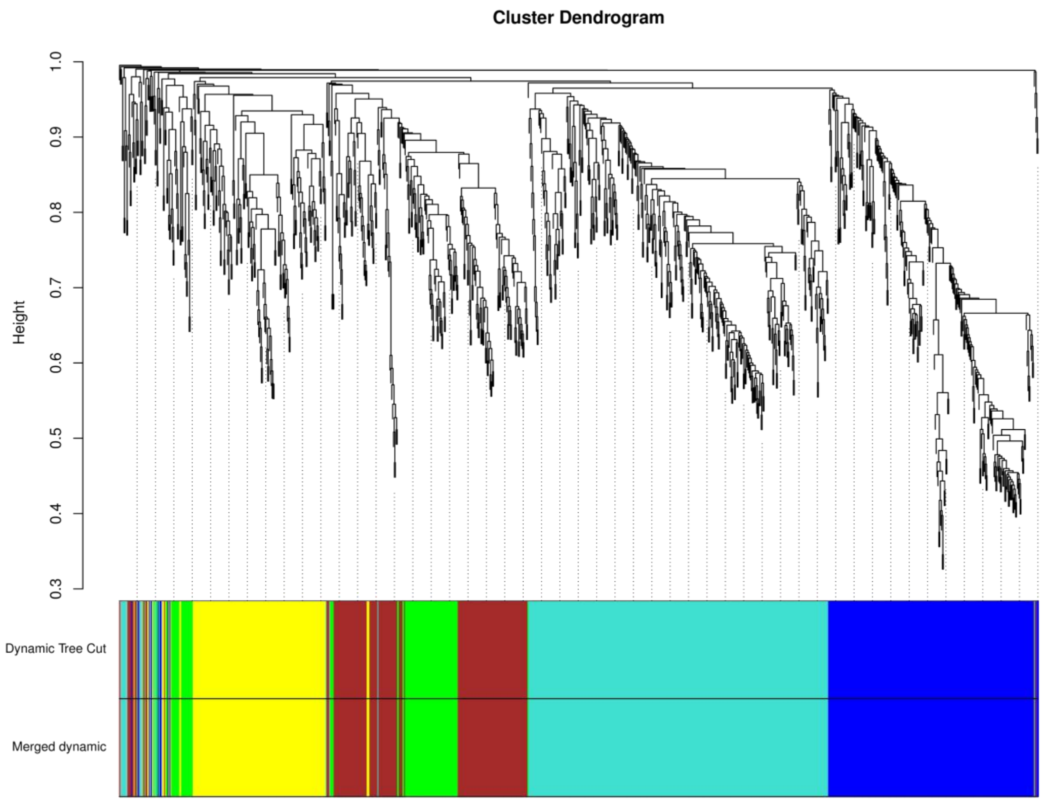

Supplementary Figure 6. Module hierarchical clustering diagram of TFs and PF metabolic pathway DEGs.
